# Genomic determinants for initiation and length of natural antisense transcripts in *Entamoeba histolytica*

**DOI:** 10.1038/s41598-020-77010-4

**Published:** 2020-11-19

**Authors:** Damien Mornico, Chung-Chau Hon, Mikael Koutero, Christian Weber, Jean-Yves Coppee, Marie-Agnes Dillies, Nancy Guillen

**Affiliations:** 1 Unité Biologie Cellulaire du Parasitisme, Institut Pasteur, Paris, France; 2 Institut National de La Santé Et de La Recherche Médicale, INSERM U786, Paris, France; 3 Plate-forme Transcriptome et Epigénome, Institut Pasteur, Paris, France; 4 Hub de Bioinformatique et Biostatistique – Département Biologie Computationnelle, CNRS USR 3756, Institut Pasteur, Paris, France; 5 Centre National de La Recherche Scientifique, CNRS ERL9195, Paris, France; 6 Present Address: Laboratory for Genome Information Analysis, RIKEN Center for Integrative Medical Sciences, 1-7-22 Suehiro-cho. Tsurumi-ku, Yokohama, 230-0045 Japan

**Keywords:** Parasite genomics, Transcriptomics

## Abstract

Natural antisense transcripts (NAT) have been reported in prokaryotes and eukaryotes. While the functions of most reported NATs remain unknown, their potentials in regulating the transcription of their counterparts have been speculated. *Entamoeba histolytica*, which is a unicellular eukaryotic parasite, has a compact protein-coding genome with very short intronic and intergenic regions. The regulatory mechanisms of gene expression in this compact genome are under-described. In this study, by genome-wide mapping of RNA-Seq data in the genome of *E. histolytica*, we show that a substantial fraction of its protein-coding genes (28%) has significant transcription on their opposite strand (i.e. NAT). Intriguingly, we found the location of transcription start sites or polyadenylation sites of NAT are determined by the specific motifs encoded on the opposite strand of the gene coding sequences, thereby providing a compact regulatory system for gene transcription. Moreover, we demonstrated that NATs are globally up-regulated under various environmental conditions including temperature stress and pathogenicity. While NATs do not appear to be consequences of spurious transcription, they may play a role in regulating gene expression in *E. histolytica*, a hypothesis which needs to be tested.

## Introduction

*Entamoeba* is a genus of unicellular eukaryote *Amoebozoa* that separated from animals and fungi lineages after the evolutionary divergence of plants. *Entamoeba* is devoid of mitochondria and is found in a wide range of animal hosts, including humans that are natural hosts of at least eight well-known species of *Entamoeba*^1^. *Entamoeba histolytica* is the amoeba parasite responsible for human amoebiasis, one of the major neglected infectious disease affecting millions of people worldwide^2^. In recent years, genomics research has led to the major advances in the understanding of the parasite and the disease. The genome of *E. histolytica* consists of a large number of repetitive elements (~ 19%) and has high AT content (~ 76%)^3^, rendering its complete assembly problematic. The current assembly is sized 20 Mbp, consists of 1498 scaffolds with 8201 predicted genes, of which the vast majority (~ 76%) does not contain introns^4^. In addition to the scarcity of intron, amoeba genes have relatively short open reading frames (ORF) (averaged ~ 389 amino acids), as compared to other unicellular eukaryotes, e.g. *Dictyostelium discoideum* (another *Amoebozoa,* 518 amino acids) and *Plasmodium falciparum* (*Apicomplexa* parasite, 761 amino acids)^5^. Genes with short ORFs is a hallmark of compact genomes, e.g. the microsporidian *Encephalitozoon cuniculi* genome encodes 2000 genes in 3 Mbp^5^. These data highlight the compactness of *E. histolytica* genome with ~ 1 gene in 2 kb, comparing to the human genome with 1 gene in ~ 120 kb. Based on genomic and cDNA sequences, the fundamental characteristics of mRNA processing has been inferred. For example, the average introns length in *E. histolytica* is 75 nucleotides with well-preserved splicing sites 5′-GUUUGUU—UAG 3′^4,6,7^ and the sequence motif involved in mRNA polyadenylation includes an AU-rich motif within the consensus sequence UA(A/U)UU^8^.

Analysis of *E. histolytica* transcriptome using RNA sequencing (RNA-Seq) suggested that 98% of the annotated coding regions is transcribed^9^. Despite the functional annotations of *E. histolytica* genome remains incomplete, several studies have used transcriptomics to understand changes in gene expression in various processes: (1) during amoebic differentiation into cysts^10^, (2) according to different degrees of parasite virulence^11–13^ or (3) as a result of trophozoite growth under various environmental constraints^14,15^. Based on these transcriptome data, several bioinformatics and experimental approaches successfully brought insight into activities in the amoebic genome. For example, diverse gene promoters have been identified regulating cell growth^16^, cyst formation^10^ or transcription during infection^17^. Recently, the correlative analysis of the genome and of the mRNA revealed several repetitive short nucleotide sequences of DNA which, according to their number of copies, modulate the expression of genes^18^. Splicing sites and polyadenylation sites on mRNA have been also characterized at the genome-wide level^19^.

The recent advances in high-throughput sequencing technologies revealed the pervasive transcription from eukaryotic genomes, producing a wide varieties of RNA transcripts which do not coding for proteins (i.e. non-coding RNAs). However, the functionality of non-coding transcripts is, to date, subject to intense debate, determining whether these RNAs are simple products of transcriptional noise or they contribute to the regulation of genetic expression^20^. Analyses of eukaryotic genomes have also identified a surprisingly high proportion of overlapping gene pairs, which often involves a long non-coding RNA (lncRNA) transcribed from the opposite strands of another gene, termed natural antisense transcripts (NAT)^21^. Despite the prevalence of NATs, its regulatory potential on gene expression has been demonstrated only in a few cases^22^. Very little is known about these molecules in *E. histolytica,* although short noncoding regulatory RNAs have been described, including small antisense RNAs^23^ and miRNAs^24^. In addition, an lncRNA has been identified in *E. histolytica*^25^, which is a polyadenylated transcript of 2.6 kb, transcribed by RNA polymerase II. Oxygen and heat stress increase expression levels of EhslncRNA indicating that it acts as general stress regulator. Given the compactness of *E. histolytica* genome, the transcribed lncRNAs, if any, are likely to overlap with another gene on the opposite strand, and their regulatory potential is worth exploring. To better understand the mechanisms of gene regulation in such a compact genome, we mapped transcript fragments, the transcription start sites (TSS) and polyadenylation sites (PAS) genome-wide and identified NAT pairs. We then quantified changes in the expression levels of NAT pairs in trophozoites harvested under growing conditions, environmental changes and during infection, aiming to explore the regulatory potential of NATs. Our data revealed the pervasiveness of NAT in *E. histolytica*, suggest that NAT transcription is not likely to be the sole consequence of spurious transcription and show that genomic sequences for TSS and polyA sites are similar for sense and antisense transcription. These facts indicate a transcriptional dynamic hitherto unknown in *E. histolytica*.

## Results

### Antisense transcription is abundant in *E. histolytica*

To characterize the transcriptome of *E. histolytica*, we sequenced poly(A) + RNA of strain HM1:IMSS (n = 1 × 4, dataset 1A) (Table S1) and performed de novo assembly of transcriptome. We obtained 27,139 contigs (i.e. transcribed fragments) with a mean length of 327 nt (from 100 to 3881nt). About 63% of these transcribed fragments (n = 16,993, average 382nt) mapped to the same strand in 81% of genes (n = 6667), attesting a good coverage of the transcriptome. Another 14% of transcribed fragments (n = 3658, averaged 222nt) mapped to the opposite strand in 28% of the genes (n = 2335). We thus conclude at least a quarter of genes showed evidence of NAT transcription (Table S2). The validation of the detected NATs was performed through a northern blot for transcribed fragments that mapped to the opposite strand of one gene (EHI_036570). We detected two NATs at this locus (after mapping the TSS and PAS, described below)*,* which both were confirmed in the northern blot with expected molecular sizes (Fig. S1).

### NATs are biased towards the 3′end of their sense counterparts

To understand the origin (i.e. biogenesis) of NATs in *E. histolytica*, we examined the overlap between annotated coding gene pairs on opposite strands. First, based on genome annotation, there were only 66 overlapping genes (Table S3). Second, only 2% of the NAT fragments (n = 73 of 3666) could be mapped to an adjacent gene on the same strand. This suggested that the majority of antisense transcription is not the consequence of read-through from adjacent genes. The contribution of overlapping coding gene pairs to the observed NATs in *E. histolytica* was rather negligible.

Several studies reported that antisense transcription could be correlated to sense gene splicing^26^. We investigated the association between antisense transcription and RNA splicing in *E. histolytica.* One-quarter of amoeba genes were predicted to be spliced (n = 2007) for a total number of 2559 of introns. By focusing on antisense transcription, we detected 151 introns (from 144 genes) overlapping, at least partially, with a NAT contig (example Fig. S2). Although splicing regulation by NAT could not be excluded, it did not seem to be a major control process. Indeed, approximatively 7% intron harboring genes were associated with antisense transcription.

**Figure 1:**
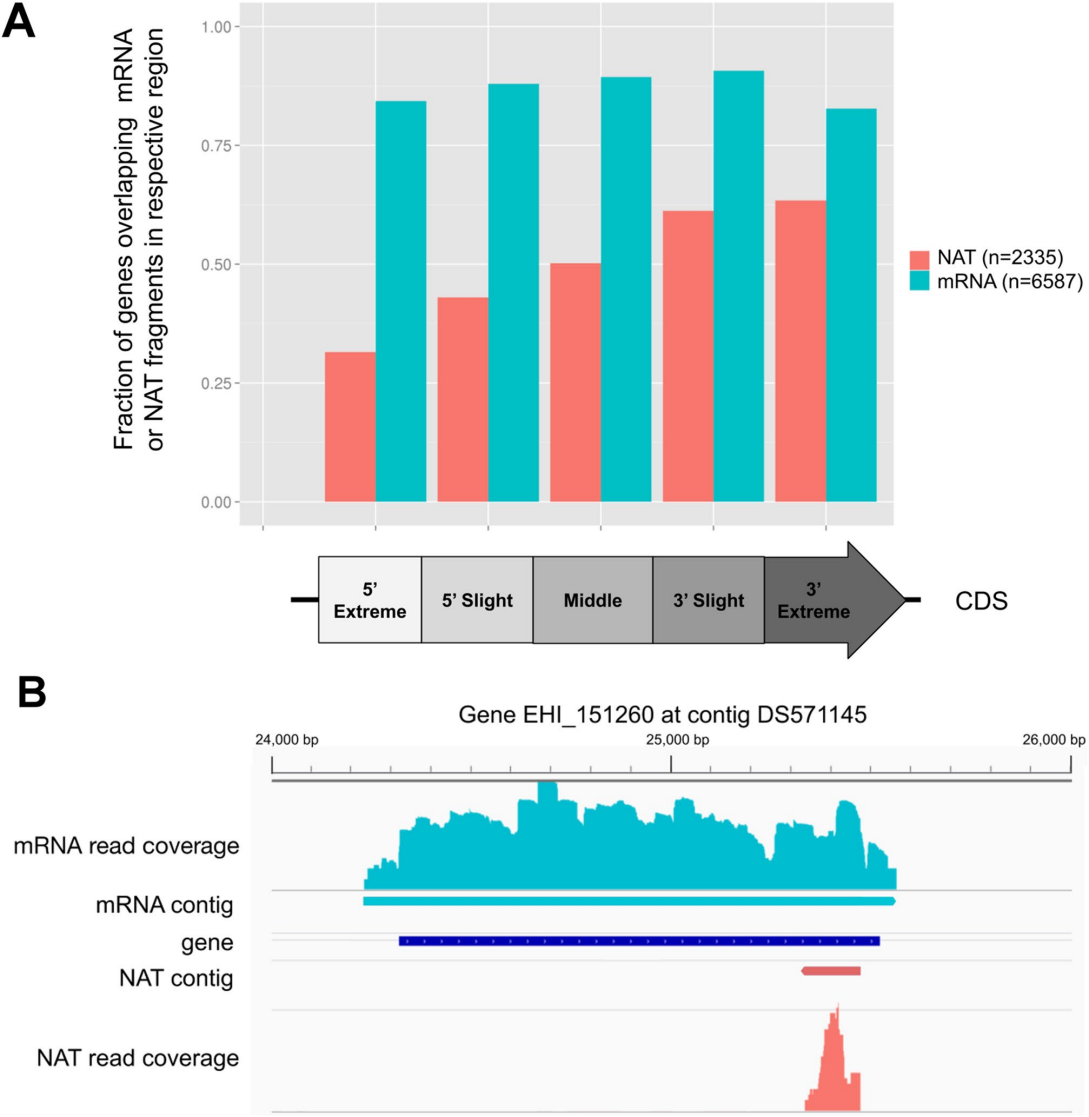
NATs and CDS overlapping patterns. (**A**) Proportion of genes overlapping at least one mRNA (blue) or NAT (red) contig (n = 6667 and n = 2335 respectively) in the different regions of their CDS on both sides. 4257 genes were identified as fully covered by mRNA fragments and 283 by NAT. (**B**) Integrative Genome Viewer (IGV, https://software.broadinstitute.org/software/igv/)^71^ screenshot for gene EHI_15260 take as an example, representing RNA-seq coverage and assembly contigs for both sense (blue) and antisense (red) transcription.

To shed light on the biogenesis of NATs, we investigated their pattern of coverage relative to their sense counterpart. We first divided the genomic regions of each coding gene into 5 equal sized bins and then counted the number of bins being covered by transcribed fragments on both strands. About 64% of the detected genes (n = 4257 of 6587) were fully covered (i.e. in all 5 bins) by transcribed fragments on the sense strand (i.e. mRNA), suggesting the de novo reconstructed transcriptome was reasonably well covered. We found only 12% of the NAT-possessing genes (n = 283 of 2335) were fully covered by transcribed fragments on the antisense strand (i.e. NAT). Most NATs were thus likely to cover partially their sense counterpart. In fact, we observed a bias of NAT coverage towards the 3′ of its counterpart, with only ~ 30% of genes covered at 5′end comparing to ~ 60% of genes covered in 3′end (Fig. 1A). Based on these observations, we concluded that most of NATs are shorter than the mRNA and their transcription initiation is biased towards the 3′end of their sense counterpart (example in Fig. 1B).

### Transcription initiation of NATs at stop codon

To get an insight into the initiation of NAT transcription, we mapped the TSS genome-wide using two libraries, one including a 5′-monophosphate dependent terminator exonuclease (TEX) (*n* = 1, dataset 2A in Table S1 and a capped small RNA (csRNA) method (*n* = 1, dataset 2B in Table S1). Briefly, the TEX method^27^ relies on the fact that 5′ ends of primary transcripts which lack a 5′-monophosphate are protected from TEX degradation, while the csRNA method^28^ is based on the fact that csRNAs (30 to 50nt) are produced at TSS through promoter-proximal pausing of Pol II^29,30^. While the mapping of a TSS of a transcript depends on its expression level in the TEX method, the level of Pol II pausing at the TSS (i.e. number of csRNA) is not strictly correlated to its expression level^30^. Therefore, these two methods are complementary. To control the level of background noise (i.e. false positive TSS derived from incomplete TEX digestion), we sequenced a control library with the same input RNA as in the TEX library but without TEX and TAP digestion, which should be unable to capture the TSS (*n* = 1, dataset 2C in Table S1). Bckground noise in both TEX and csRNA libraries were then subtracted, based on the ratio of reads in TEX or csRNA libraries to the control library (see “Methods”).

**Figure 2:**
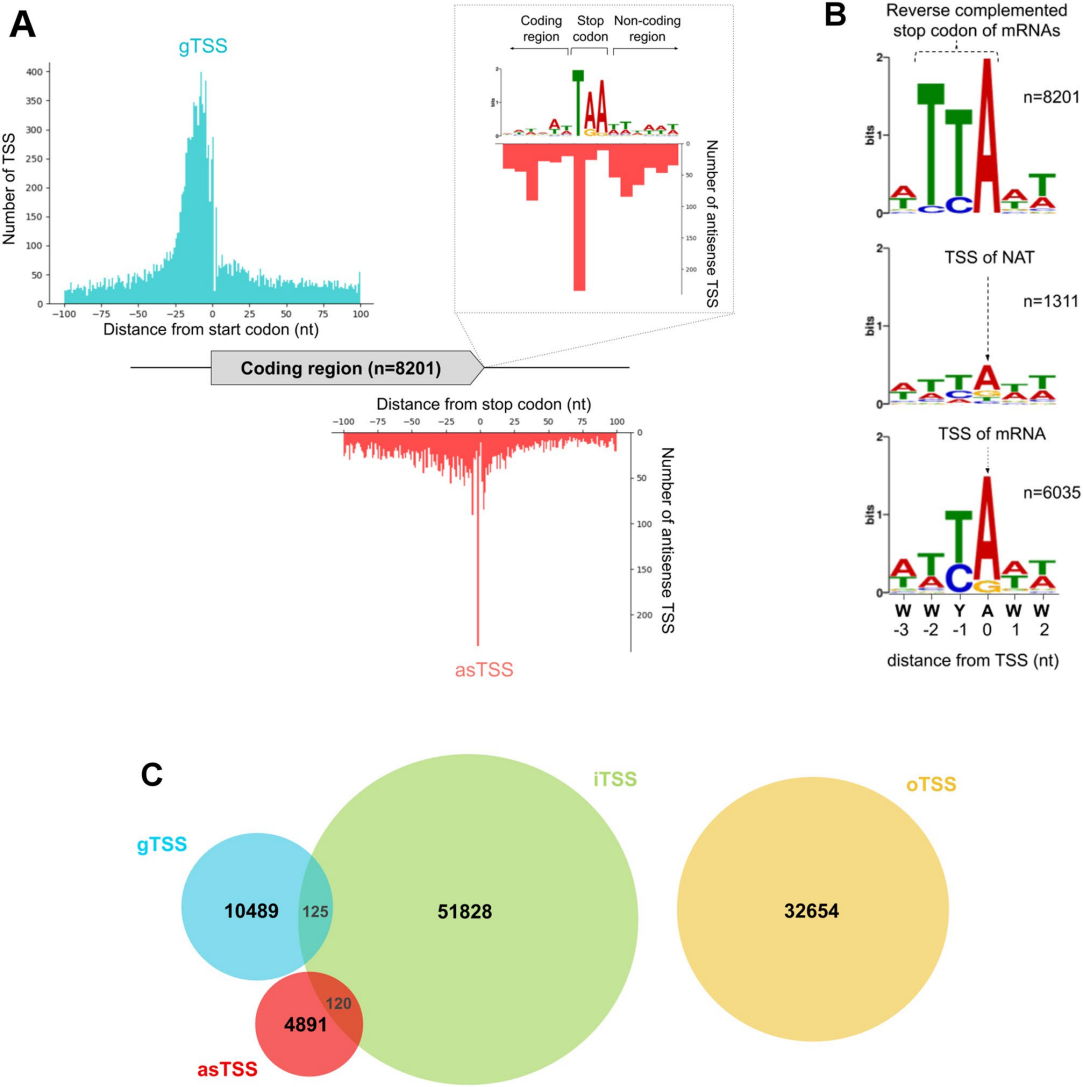
Transcription Start Sites identification for mRNA and NAT. (**A**) Mapping of TSS at CDS boundaries (with 100nt apart) on both strands, the region in the vicinity of the stop codon is magnified in the insert of the upper right corner. (**B**) Sequences logo computed around stop codon using the entire genome CDS reverse complemented (upper panel). asTSS (middle panel) and mRNA TSS (lower panel) found in this work. (**C**) Number of each type of TSS.

We then pooled the background subtracted signal (i.e. candidate TSS) from both TEX and csRNA dataset and clustered these candidate TSS (see “Methods”), yielding 95,326 TSS (i.e. TSS clusters, Table S4). These TSS were strongly enriched around the annotated start codons, peaking at 10nt upstream (Fig. 2A). This observation was consistent with the small size of intergenic regions (Fig. S3) and previous findings on the relative short 5′UTR in *Entamoeba* (~ 5 to 20 nt)^31,32^. Overall, the employed method has resulted in a successful and consistent capture of the TSS of mRNAs. In addition, about 73% of genes (n = 6035 of 8201) exhibited at least a TSS mapped within 100nt upstream of its start codon, suggesting a reasonable coverage of our TSS mapping. On the antisense strand, we observed a sharp peak of TSS surrounding the stop codon (Fig. 2A), consistent with the bias of NAT coverage towards gene 3′end as discussed earlier (Fig. 1).

Based on these observations, we classified all TSS into 4 classes (see “Methods”): 1) gTSS: “gene TSS” at proximity of start codon (100nt upstream) on the sense strand (n = 10,614 in 6035 genes), 2) asTSS: “antisense TSS” close to stop codons (+ /– 100nt) on the antisense strand (n = 5011 in 2972 genes), 3) iTSS: “internal TSS” within the ORF on same strand ( n = 52,073 in 6654 genes) and 4) oTSS: “orphan TSS” in between genes ( n = 27,873) (Fig. 2C). These oTSS may either correspond to unannotated ORFs located at contig boundaries of the draft genome^33,34^, or to possible artifacts. They were not further considered in the following analysis.

About 54% of the NAT-possessing genes (n = 1311 of 2335) have at least one asTSS, implying at least a half of observed NAT was initiated within a 200nt window surrounding the stop codon (defined as terminal-associated NAT, ta-NAT). Thus, the stop codon represents a hotspot for antisense transcription initiation, and we indeed observed a sharp peak of TSS precisely located at the first base of the stop codon (Fig. 2A). To understand the genomic determinants for transcription initiation, we investigated the initiator motif (Inr) at the TSS of both mRNA and ta-NAT^31^. Analyses of TSS peak sequences revealed a conserved RNA Polymerase II (Pol II) Inr^31^ for both mRNA and ta-NAT (i.e. WWYAWW, Fig. 2B), suggesting the majority of ta-NATs are transcribed by Pol II and the criteria for local selection of TSS by Pol II is similar in both mRNA and ta-NAT. To explain the observed preference of ta-NAT TSS at the stop codon (Fig. 2A), we compared the sequence around the stop codon (reverse complemented), with the Inr sequence of mRNA and ta-NAT (Fig. 2B). The reverse complement of the 6 nucleotides surrounding the stop codon (Fig. 2B upper panel), resembled the Inr motif (Fig. 2B middle and lower panel). In particular, the first base (i.e. T) and second base (i.e. T or C) of the stop codon was similar to the YA dinucleotide at –1 and 0 position of Inr motif. The ta-NAT TSS hotspot at stop codon might thus be a consequence of preferential TSS selection by Pol II, due to the Inr motif at stop codon on the antisense strand.

### NAT and mRNA share the same core promoter architecture

**Figure 3:**
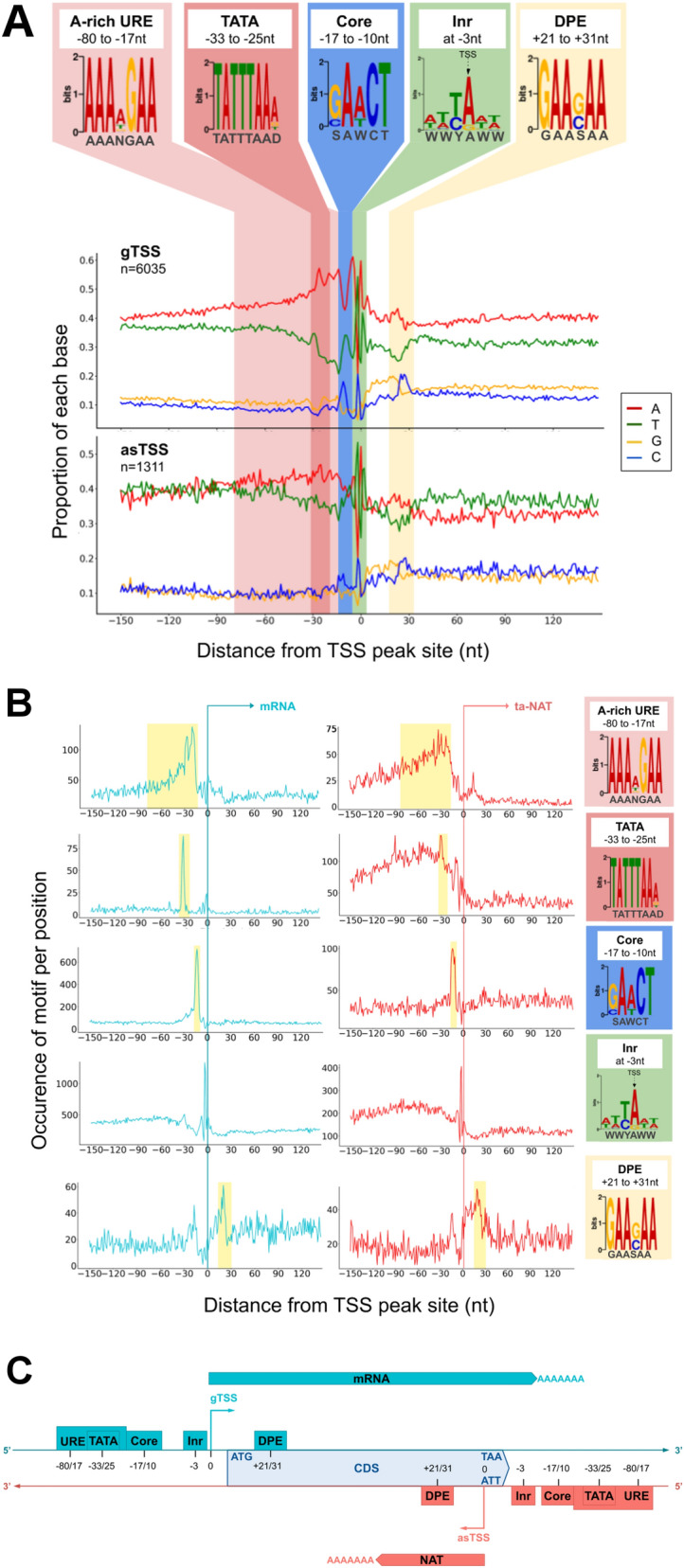
Transcription Start Sites motifs enrichment for mRNA and NAT. (**A**) Nucleotide sequence composition and motifs enrichment around TSS of both mRNA and NAT. When several are assigned to a same gene, only the strongest TSS is considered (primary). Notice that 0 corresponds to the TSS peak identified (**B**) Appearance frequencies of each motif around TSS of both mRNA and NAT. (**C**) Schematic representation of TSS promoters positions on both DNA strands.

We compared the sequence composition surrounding the TSS of mRNA (gTSS) and ta-NAT (asTSS) and found that they share similar characteristics (Fig. 3A), including (1) an A-rich region around − 80 to − 20 nt, (2) a C/T enriched region around − 10 nt, (3) a YA motif around TSS (i.e. Inr in Fig. 2B), as well as (4) a C/G enriched region at + 25nt. In addition, a T/A enriched region (at − 30 nt) was found within the A-rich region in gTSS, and to less extent in asTSS. Motif enrichment analyses of gTSS sequences revealed the overrepresentation of 4 motifs (in addition to Inr in Fig. 2B), including an A-rich Upstream Regulatory Element (i.e. A-rich URE, *AAANGAA, p* = *5.5e−044*), a TATA-like box (i.e. TATA, *TATTTAAD, p* = *9.9e−050*), an upstream core motif (i.e. Core, *SAWCT, p* = *2.7e−588*) and a downstream promoter motif (i.e. DPE, *GAASAA, p* = *1.7e−019*) (Fig. 3A). It is noted that the TATA, Core and Inr described here are likely homologous to the previously described “non-consensus” TATA element, GAAC element, and initiator element respectively^35^. While the A-rich URE might be homologous to some of the “UREs” described previously^35^, in this study, it refers to a loosely conserved A-rich motif broadly distributed between − 80 to − 20 nt, and the TATA-like box seems to be a specific variant of A-rich URE located sharply at − 30 nt. The DPE described here is likely to be homologous to that of promoters in *Drosophila melanogaster*^36^. Analyses of motif occurrence showed that all of the 5 motifs were equally positionally enriched in both gTSS and asTSS (shaded ranges in Fig. 3B). These results clearly suggest that NAT and mRNA transcription takes place from core promoters with the same genomic architecture (Fig. 3C).

### Genomic determinants for polyadenylation and length of NATs

**Figure 4:**
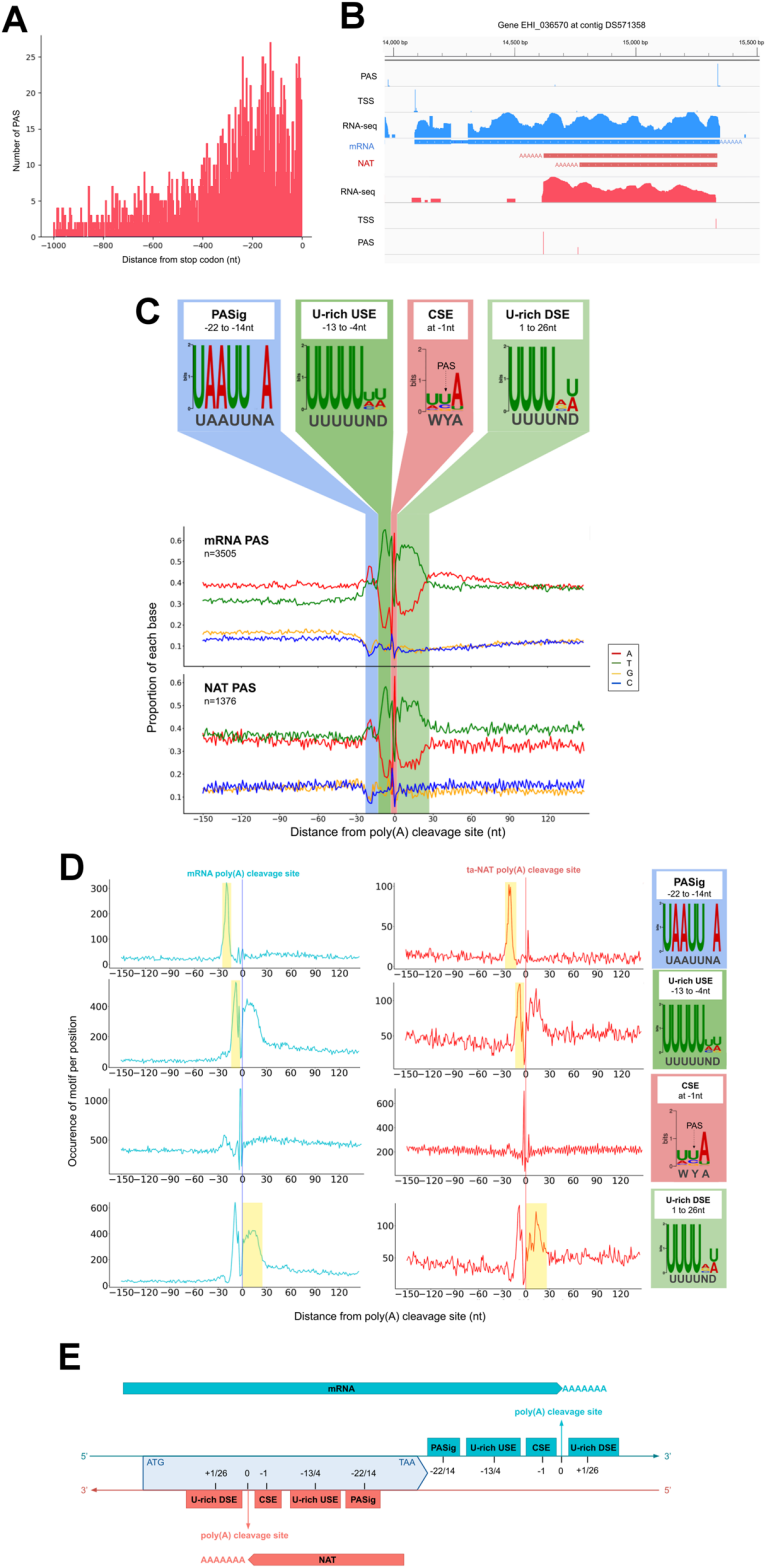
Polyadenylation sites features for mRNA and NAT. (**A**) Distribution of PAS distance from stop codon (position 0) on antisense DNA strand. (**B**) Integrative Genome Viewer (IGV, https://software.broadinstitute.org/software/igv/)^71^ screenshot for gene EHI_036570, representing RNA-seq coverage, TSS and PAS on both direct and reverse strand. (**C**) Nucleotide sequence composition and motif enrichment around the RNA cleavage site (position 0 corresponds to 1nt before the cleavage site) of both mRNA and NAT. (**D**) Appearance frequencies of each motif around the RNA cleavage site of both mRNA and NAT (position 0 corresponds to 1nt before the cleavage site). (**E**) Schematic representation of PAS patterns positions on both DNA strands.

As the above libraries were prepared from polyadenylated RNAs, we assumed the observed ta-NAT is polyadenylated. Next, we sought to investigate the genomic determinants of PAS of ta-NAT. We mapped the PAS genome-wide using the method we described previously^19^. Majority of identified PAS are located 20nt downstream of the stop codon, corresponding to the expected location of mRNA PAS^19^, suggesting most of the identified PAS are genuine. Closely located PAS were grouped into clusters as described previously^19^. Information of all PAS (n = 6518) can be found in Table S5. Totally 2737 PAS were mapped to the antisense strand within the ORF of 1996 genes (Table S6). About 60% of these antisense PAS (883 of 1493, only genes with ORF > 1 kb were considered, Fig. 4A, Table S5) are located within 500 bp upstream of the stop codon, suggesting the majority of ta-NATs are polyadenylated within short distance after their transcription initiation from the 3′end of genes, generating relatively short ta-NAT transcripts. In some cases, we observed multiple antisense PAS within 1 kb upstream of stop codon (n = 523 genes, Table S6), generating short ta-NATs of various lengths (an example in Fig. 4B, northern blot confirmation in Fig. S1). These data suggest, to some extent, ta-NATs could be multiple overlapping short transcripts initiated from the same TSS at gene 3′end, but ended with various lengths, depending on the choice of PAS.

Our previous analyses showed the conserved motifs surrounding PAS of eukaryotic mRNA are generally present in *E. histolytica* mRNA^19^. In fact, the sequence compositions surrounding the PAS of mRNA and ta-NAT were similar (Fig. 4C), suggesting these motifs are also present in PAS ta-NAT. Motif enrichment analysis suggested an overrepresentation of 3 motifs surrounding the PAS, (1) Polyadenylation Signal (PASig, UAAUUNA, *p* = 1.1e*−*238), (2) U-rich Upstream Sequence Element (U-rich USE, UUUUUHD, *p* = 1.1e*−*574) and (3) U-rich Downstream Sequence Element (U-rich DSE, UUUUNW, *p* = 6.2e*−*478). We then defined a Cleavage Sequence Element (CSE, WYA) by aligning the 3 nucleotides around peak of mRNA PASC (Fig. 4C). The occurrence of these motifs surrounding the mRNA and ta-NAT PAS are very similar (Fig. 4D), suggesting the location of ta-NAT PAS depends on the same set of genomic determinants as those of mRNA, which are thus reverse encoded in the gene coding sequence (Fig. 4E).

Based on these results, codon might be selected in order to favor the emergence of these motifs on the opposite strand. Considering the Relative Synonymous Codon Usage (RSCU), we found that codons with an A in the synonymous position is preferentially selected against the ones with a T for a same amino acid (Fig. S4). In line with this observation, the codon usage of *E. histolytica* might be evolutionarily selected to adapt to the enrichment of U-rich motifs in opposite strand and might represent an evolutionary strategy for compact genomes to attain extra regulatory components.

### Expression of NATs is regulated upon stress and between trophozoites of different virulence

**Figure 5:**
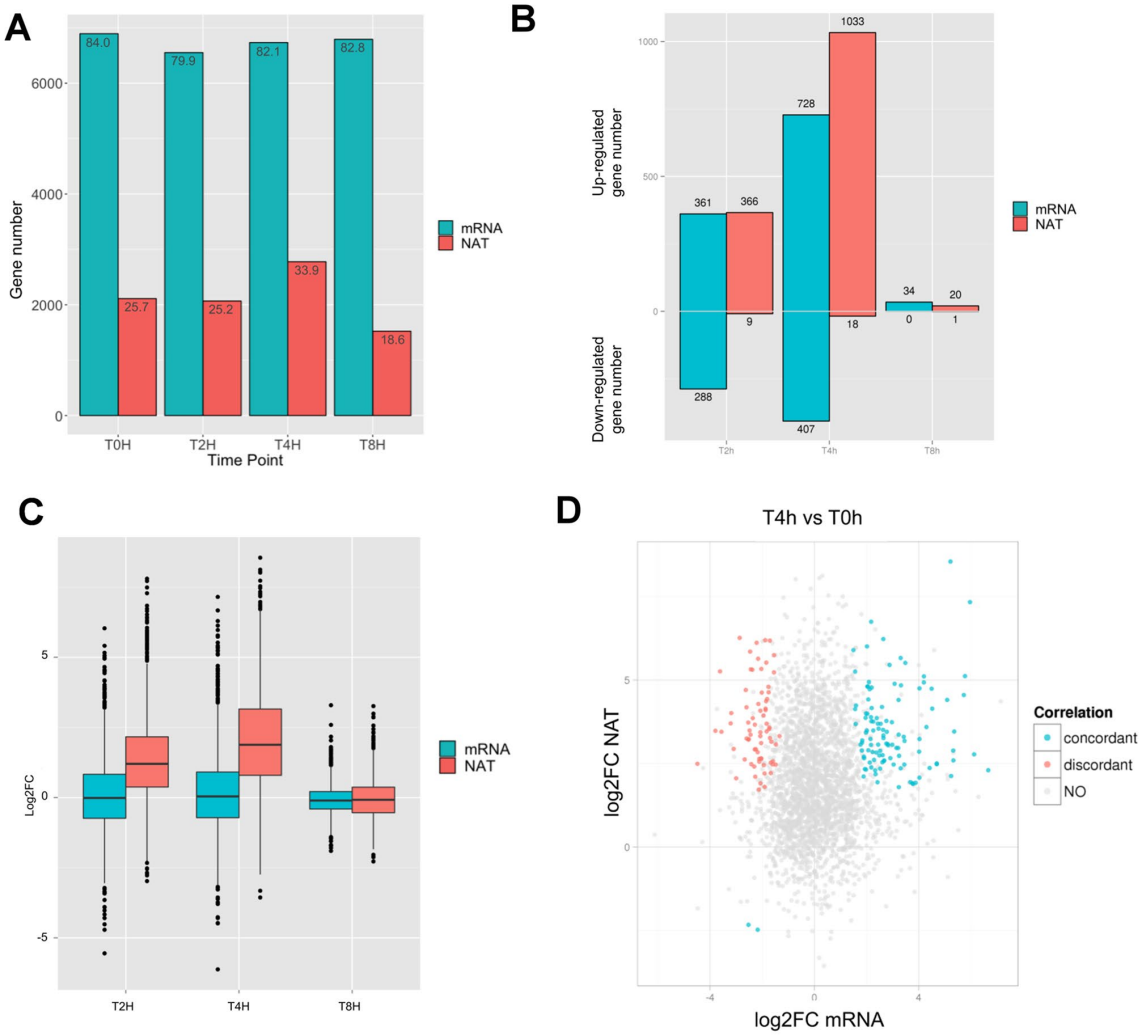
Impact of temperature change on transcription. (**A**) Number of genes overlapped by mRNA and NAT contigs at different time-points of the experiment. (**B**) Proportion of up-regulated and down-regulated mRNA and NAT at T2h, T4h and T8h versus T0h, detailed in Table S8. (**C**) Log2FC box-plot for all genes at T2h, T4h and T8h versus T0h. (**D**) The fold change in gene expression (FC) was compared by plotting the log2FC of mRNA (x axis) versus log2FC of NAT (y axis) for each gene having at least one NAT contig identified, at T4h versus T0h. The color of points illustrates the differential expression type: none or unidirectional (grey), both concordant (blue), both discordant (red). Figure produced using R^69^ and ggplot2^70^.

We assessed whether NAT expression was regulated by analyzing its transcription in different contexts. We profiled, using RNA-Seq, the transcriptomes of (1) trophozoites under stress (i.e. heat shock and recovery) and (2) trophozoites exhibiting different virulence. Here we first focus on their transcriptomes upon heat shock and recovery. Briefly, cells were passed from 37 to 42 °C, followed by a recovery stage at 37 °C. Samples were collected at 0 h (37 °C), 2 h (42 °C), 4 h (42 °C) and 8 h (37 °C) (dataset 3 in Table S1, “Methods”). We counted the number of genes with clear NAT transcription at each timepoint, by the presence of antisense transcribed fragments from de novo transcriptome assembly (Table S7, “Methods”). While the percentage of genes with mRNA transcribed fragments is relatively steady across time points (~ 79–82%), we observed a substantial increase of the percentage of genes with NAT transcribed fragments during heat shock at 4 h (~ 34%) followed by a decrease during recovery at 8 h (~ 19%) (Fig. 5A). The data suggested there was a general induction of NAT transcription under stress (during heat shock at 4 h), and the induced NAT transcription was reversible (during recovery at 8 h). This observation is consistent with differential expression analyses (“Methods”), for which we observed a sharp increased number of genes with significantly antisense transcription up-regulated (adjusted *p* value $\le$ 0.05 with at least twofold-change, “methods”, Table S8) during heat shock at 4 hr (compared to 0 hr) (Fig. 5B,C).

Then, we explored the interaction between sense (i.e. mRNA) and antisense (i.e. NAT) transcription. We wondered whether a mRNA is more likely to be differentially expressed upon heat shock (i.e. dependent) when NAT is transcribed at the same locus. Focusing on 4 h, 1135 mRNAs were differentially expressed (728 up- and 407 down-regulated compared to 0 h) and 17% of them have NAT transcribed. A ***χ***^2^ test revealed a significant, although moderate, dependence of mRNA differential expression and NAT presence (***χ***^2^ = 5.4994, df = 1, *p* = 0.0190, Table S9—sheet (1), implying a potentially general impact of NAT in regulation of mRNA expression. However, we did not observe a global correlation between the fold-changes of mRNA and NAT (R^2^ = 0.0018, Fig. 5D). Notably, there were 185 pairs of mRNA and NAT differentially expressed at the same time. 113 pairs changed in the same direction (i.e. concordant, *blue* in Fig. 5D) and 72 pairs changed in the opposite direction (i.e. discordant, *red* in Fig. 5D). Previous studies suggested the concordant (i.e. correlated) and discordant (i.e. anticorrelated) expression of mRNA and NAT pairs might have reflected different regulatory relationships with distinct molecular mechanisms, e.g. co-degradation in concordant expression and transcriptional interference in discordant expression^37^. While our data showed no systematically concordant or discordant expression between the mRNA and NAT pairs in *E. histolytica,* some genes display these peculiar patterns of transcription (Table S9—sheet 2). As examples, small GTPases; guanine nucleotide exchange factor GEF, cysteine proteases, chitinase and glycoprotein Jacob involved in encystation are among the concordant NAT- mRNA upregulated gene expression, whereas several GTPase activating protein GAP, two cysteine protease binding proteins and several enzymes are discordant.

**Figure 6:**
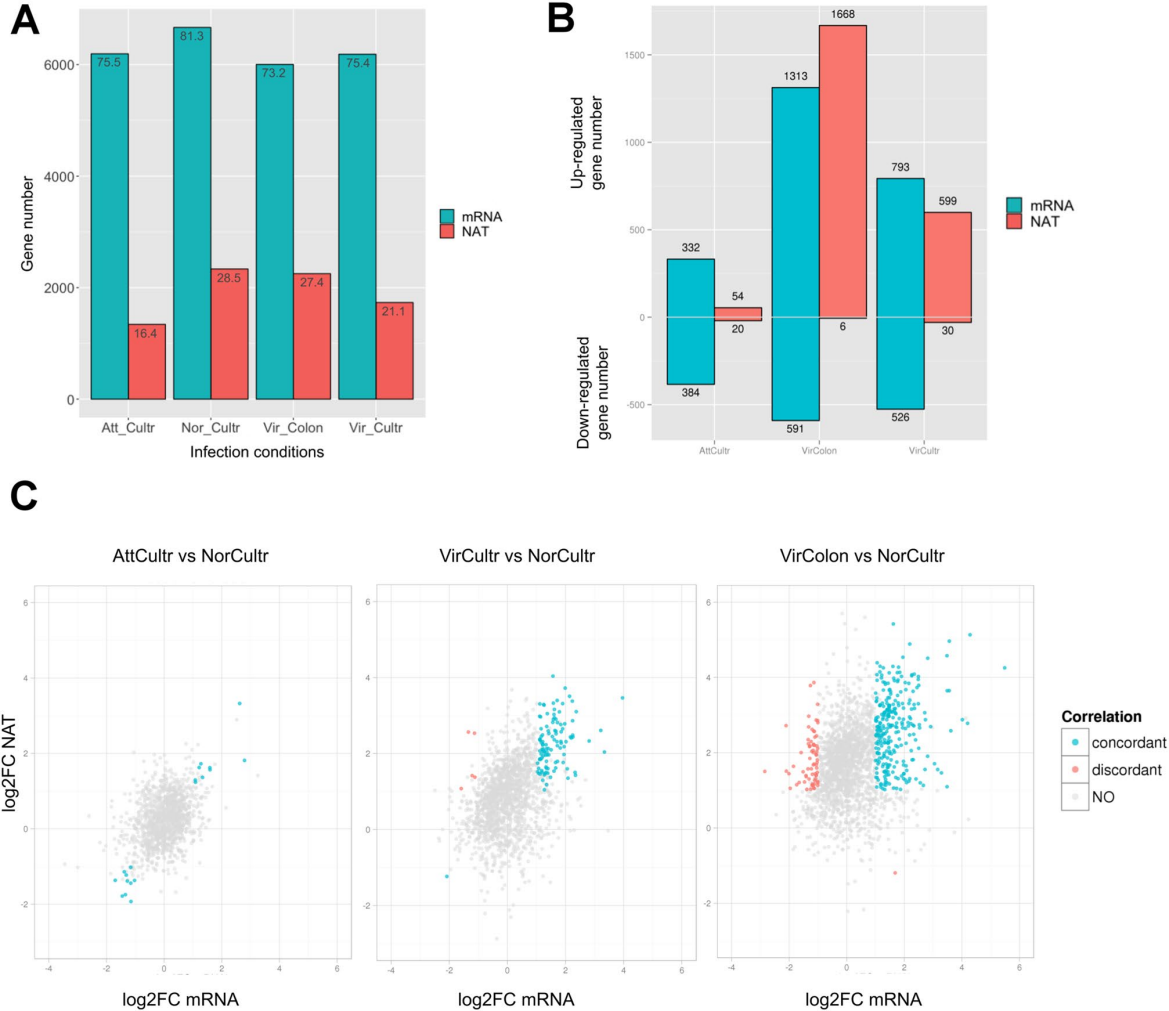
Infection condition effects on transcription. (**A**) Number of genes overlapped by mRNA and NAT contigs in different infection conditions of the experiment. (**B**) Proportion of up-regulated and down-regulated mRNA and NAT in AttCultr, VirColon and VirCultr versus NorCultr, detailed in Table S12. (**C**) The fold change in gene expression (FC) was compared by plotting the log2FC of mRNA (x axis) versus log2FC of NAT (y axis) for each gene having at least one NAT contig identified, in AttCultr, VirCultr or VirColon versus NorCultr. The color of points illustrates the differential expression type: none or unidirectional (grey), both concordant (blue), both discordant (red). Figure produced using R^69^ and ggplot2^70^.

To further extend the above observations, we performed similar analyses on the transcriptomes of trophozoites of different virulence and in different infection conditions, as described previously^12^ (dataset 1A–D in Table S1, “Methods”). Briefly, we profiled samples: (1) the “normal” trophozoites maintained in short-time culture (NorCultr), (2) virulent trophozoites freshly extracted from hamster with liver abscess (VirCultr), (3) virulent trophozoites cultured in human colon explants (VirColon), (4) attenuated trophozoites with long-time culture (AttCultr). First, we observed slight changes of NAT transcription across the 4 infection conditions (in terms of presence of antisense transcribed fragments as described) (Fig. 6A, Table S10). We next identified the genes differentially expressed in VirCultr, VirColon and AttCultr, compared to NorCultr (Fig. 6B, Table S11). The independence of both sense and antisense transcription, was tested using a ***χ***^2^ test based on the presence or the absence of associated NAT with differentially expressed or not modulated genes (Table S11). To refine this relation, we performed separately two analyses on differentially up and down expressed genes to figure out if the dependency was more significant in up or down regulation. We highlighted a dependence between presence of NAT and the up regulation, specifically in “Vir” and “VirColon” conditions (*p* = 7.925e−05 and *p* = 1.48e−02 respectively). Even if we could not exclude a dependency between NAT presence and up-regulation of mRNA in some genes due to a general increase of transcription on both strands, some regulatory mechanisms have already been demonstrated for common upregulation of transcripts sense and antisense^38^. Next, similar to the observations in heat shock, we did not observe a global trend of changes in mRNA and NAT co-expression, as illustrated in Fig. 6C. The majority of identified genes present a concordant pattern (Table S11, sheet 2), whereas was noticeable 71 genes in the condition VirColon versus NorCultr presenting a discordant pattern (NAT up-regulated and mRNA down-regulated); among them several enzymes can be highlighted (Table S11, sheet 3).

Finally, we identified a set of genes (n = 457) exhibiting a significant NAT transcription in all 8 samples described above (Table S13, sheet 1). Among them 257 gene products were identified at UniProt library collection and corresponds to proteins grouped in 6 major categories including: cytoskeleton related; traffic, signaling and stress; enzymes; nucleic acid interactors and pathogenesis (Table S13, Sheet 1). There we highlighted proteins having a role in pathogenesis such as the Gal/GalNAc lectin intermediate subunits Igl1 and Igl2 (EHI_006980, EHI_065330) and the two light chain subunits EHI_148790 and EHI_058330. The amoebapore B, two proteinases, the serine rich protein, the 20 kDa factor and 10 members of the BspA family were also identified. Moreover, 93 identified genes are full length covered by NAT in trophozoites under normal culture conditions (Table S13, sheet 2).

## Discussion

Antisense transcription is a general feature of living entities and cells since it has been reported in viruses^39^, bacteria^40^, protozoa^41^, fungi^42^, plants^43^, invertebrates^44^ and mammals^45^. Although the proportion of the observed NATs being functional remains elusive, a number of NATs are reported to modulate the expression of their cognate sense RNA through various mechanisms (for reviews see^22,46^). For example, such a modulation may arise epigenetically, through recruitment of Polycomb Recessive Complex 2 for gene-silencing by histone modification, or transcriptionally, through formation of RNA: RNA duplexes that affects alternative splicing, nuclear retention, RNA editing and transcript stability. Here we demonstrated that more than a quarter of the genes in *E. histolytica* showed significant evidence of NAT expression, which is far from negligible compared to other species^47^ and equivalent to the proportion of NATs found in humans^48^. For instance, few NATs have been identified in *Dictyostelium discoideum*^49^ and *Trypanosoma brucei*^50^ (162 and 182 pairs respectively), while *Plasmodium falciparum* shows a similar ratio of antisense transcription (24% of protein-coding genes)^41^.

Based on genome-wide TSS mapping, we demonstrated the majority of *E. histolytica* NATs are transcribed around the 3′ end of the ORFs, and in particular precisely initiated at the stop codon with recognizable promoter architecture. In addition, by genome-wide PAS mapping, we showed these NATs are polyadenylated and their PAS are reversely encoded in the corresponding ORF which has a deterministic effect on the length of NATs. These results suggest the majority of the observed NATs are not likely to be due to sequencing artefacts. More importantly, the encoding of NAT regulatory sequences (i.e. motifs associated with their TSS, PAS and Inr) on the opposite direction of the encoding DNA strand, unambiguously indicates for the first time the highly efficient usage of intergenic spaces in such a compact genome.

Interestingly, we observed a global up-regulation of NAT transcription during heat shock back to normal levels during recovery, suggesting the expression of NAT is physiologically regulated, despite their potential roles in regulation of gene expression remains elusive. The significant over-representation (***χ***^2^ test, *p* < 0.05) of NAT in genes with differentially expressed mRNAs implies some potential mechanisms of co-regulation, or co-biogenesis, between NAT and mRNA pairs. Intriguingly, we did not observe a global correlation, or anti-correlation, between the expression of NATs and their cognate mRNA. This lack of global correlation is similar to the NATs observed in *P. falciparum*^41^. Nonetheless, we cannot exclude the presence of distinctive and heterogeneous mechanisms^38,47,51–53^ underlying the above observations, as we noticed some genes exhibiting clear concordant or discordant transcriptions. This study identified a set of 457 genes with confident NAT transcription across multiple experiments, representing a great opportunity for further experimental and in silico approaches to interrogate their roles in gene regulation, particularly for the ones associated with *E. histolytica* virulence, such as the Gal/GalNAc lectin subunits.

A strong correlation between the percentage of A/T bases of a genome and the number of identified antisense RNAs was demonstrated^54^. The high frequency of A/T increases the random occurrence of core promoter motifs, which are enriched with A/T. One could therefore speculate that NATs identified in *E. histolytica* (whose genome bear 76% of A/T bases) are largely derived from aleatory promoters motifs, and antisense transcription might largely represent noise. However, we argue that their physiologically regulated expression under heat shock disadvantage this hypothesis. Also, while a wide distribution of TSS is expected if the emergence of NAT is random, instead, we observed a sharp TSS peak on stop codon. All the more so as this particular region has already been identified as a potential target for antisense regulation mechanism^55^. Overall, we concluded that paired with the compact genome constraints above discussed, these NATs offer great embedded opportunities for transcription regulation to *E. histolytica*.

## Methods

### Culture of Entamoeba histolytica

*Entamoeba histolytica* strain HM1:IMSS was cultured in TYI-S-33 medium at 37 °C^12^.

### RNA isolation and northern blot analysis

Trophozoites growing in TYI-S-33 medium were harvested and total RNA extracted using TRIZOL reagent (Invitrogen) and cleaned using the RNEasy cleanup kit (Qiagen). Northern blots were performed according to the following protocols. Briefly, 10 μg or 20 μg of total parasite RNA (per electrophoresis lane) was denatured at 65 °C, loaded and molecules separated in a 1% agarose gel electrophoresis for 2 h at 95 V. RNA size standards were used to calibrate the samples (Ambion ref. 7170). RNA was transferred during 2 h to membrane filters (Ambion Bright star, ref. AM10102), the filters were crosslinked to the membrane by exposition to 1200 uj and hybridized with biotinylated probes.

To prepare the RNA probes, DNA fragments of different lengths were PCR amplified from the amoebic genome according to EHI_ 036570 gene sequence, using diverse primers according to the gene sequence and adding a T7 primer for in vitro amplification of the RNA (Fig. S1). PCR conditions using 1 µg of genomic DNA were: 94 °C for 3 min followed by 32 cycles of 94 °C for 30 s, 54 °C for 30 s and 72°c for 45 s and then the PCR product was incubated for 5 min at 72 °C. In vitro transcription using biotinylated nucleotides and PCR fragments was done in the presence of T7 polymerase and Bio-11-UTP (Ambion ref. AM845) according to furnisher protocole (Ambion ref. AM 2082). The RNA product was treated by Turbo DNAse, precipitated by ethanol and resuspended in distilled water, quantified with a NanoDrop and used for northern blotting experiments above described. RNAs were detected using the BrightStar BioDetect Kit (Ambion ref. AM1930) with prehybridization at 65 °C for 30 min, hybridization overnight at 62 °C and chemiluminescent alkaline phosphatase substrate revelation with the CDP-Star Substrate (ThermoFisher ref. T2145). Blots were exposed to film, subjected to autoradiography, scanned, and prepared for publication using Adobe Photoshop (version 7, San Jose, CA).

### Search for gene pairs in the genome of E. histolytica

To examine the eventual presence of gene pairs on the genome of *E. histolytica* HM1: IMSS, we used the colocation tool from AmoebaDB (https://amoebadb.org/amoeba/). The steps were as follows: search for all genes in Taxonomy/organism (get an answer), click on Add a step, search for Taxonomy/organism and search and choose 1 relative to 2 in the overlapping diagram to combine the search results, click on continue. In the open window organize the colocation tool: click "genes from step 1" (small window in the left) and see the text whose exact region overlaps a gene from step 2 and is on "the opposite strand" (small window in the right). Click on submit. The tool proposes the overlapping genes on a new window.

### RNA extraction, library construction and sequencing

Total RNA was extracted from approximately $1 \times 10^{6}$ *E. histolytica* trophozoites (with each sample performed in triplicates) using Trizol (Invitrogen). The polyA fraction was purified from 10 to 100 µg of total RNA using Dynabeads according to the manufacturer’s instructions (Thermofisher ref. 28152103011150). Libraries were constructed using ScriptSeq mRNA-Seq Library Preparation Kit (Illumina) following manufacturer's recommendations and were quality controlled using Agilent Bioanalyzer. Sequencing was performed on a HiSeq 2000 (Illumina) to obtain 58 base single-end reads. All reads from different experiments were cleaned from adapter sequences with AlienTrimmer^56^ (version 0.4.0) and low quality (< 20) or short sequences (< 20 nt) were removed from datasets.

### Genome reference and data repositories

The reference genome was downloaded from AmoebaDB v34^57^ (https://amoebadb.org/common/downloads/release-44/EhistolyticaHM1IMSS/). The total genome size is 20,80 Mbases, with a GC content of 24.2%, assembled in 1496 contigs, and 8201 annotated coding sequences. The data from experiments of TSS identification and heat-treated amoebae (dataset 2 and 3 in this paper) are available in the SRA database (https://www.ncbi.nlm.nih.gov/sra/) under the accession number PRJNA615171. The data concerning virulence conditions (dataset 1) derived from (Weber et al.)^12^, these data were already in the ArrayExpress database (www.ebi.ac.uk/arrayexpress) under accession number E-MTAB-4882.

### Transcript contig construction

Illumina libraries were merged within each dataset (Table S1) for NAT fragment analysis in order to reach bigger set of reads. Sequences were mapped to the reference genome of *E. histolytica*, using STAR v2.5.0^58^ with a maximum intron length of 900 (–alignIntronMax), 2 mismatches maximum (–outFilterMismatchNmax), 10 locations maximum for a read mapping (–outFilterMultimapNmax), minimum overhang (–alignSJoverhangMin) of 25 for spliced alignments. Mapped reads were then split into direct and reverse strand sets with SAMTools^59^. Trinity assemblies^60^ were performed on each set (direct and reverse) to construct transcript fragments, with a kmer size of 15nt (–KMER_SIZE 15), a maximum intron size of 900nt (–genome_guided_max_intron 900), a minimum coverage of 20 (–genome_guided_min_coverage 20) and a minimum length of 100nt for each assembled contigs (–min_contig_length 100). Resulting transcript contigs were mapped on the reference genome using Gmap^61^ with a maximum intron length of 900nt (-K 900). Lastly, NAT fragment counting for each gene was produced by featureCounts^62^ on the opposite strand (-s 2), with a minimum fraction of 10% of the contig length overlapping the gene, required for its assignment (–fracOverlap 0.1). Genes were counted as “NAT gene”, when at least 1 NAT contig was identified with featureCounts. In order to specify parts of genes preferentially covered by NAT contigs, we split the genes in 5 equal regions and counting was performed on each region as well, allowing contig assignment to all of their overlapping parts (-O -f).

### TSS identification and annotation

Total RNA (10–100 μg) was polyA enriched twice using Sera-Mag Magnetic Particles Oligo(dT) coated (Thermo Scientific ref. 28152103011150) and fragmented 5 min (Ambion ref. AM8740). According to manufacturer's instructions, Transcription Start Site enrichment was achieved with Terminator 5′-Phosphate-Dependant Exonuclease (TEBU ref. TER51020) followed by Tobacco Acid Pyrophosphatase (TEBU ref. T81050) treatment. For CapSeq, total RNA was similarly treated with an additional 15% TBE-urea gel size selection. RNA was treated with Terminator 5′-phosphate-dependent Exonuclease (Epicenter ref. TER51020), calf intestinal phosphatase (New England Biolab ref. M0290) and Tobacco Acid Pyrophosphatase (Epicenter ref. T19050). Libraries were constructed following manufacturer's recommendations using TruSeq Small RNA Sample Prep Kit (Illumina, ref. RS-200-0012). The libraries were purified with AMPure XP beads (Agencourt ref. A63880) and controlled by Bioanalyzer DNA High Sensitivity Chips (Agilent ref. 5065-4626). Sequencing has been performed on a HiSeq 2000 (Illumina) in a multiplexed 51 + 7 bases single read using a TruSeq SR Cluster kit v2 cBot HS (Illumina, ref. GD-401–2510) and a TruSeq SBS kit v2 HS 50 cycles (Illumina, ref. FC-401-1002). After sequencing, files were generated using CASAVA 1.8.2 (Illumina). The TSS libraries yielded a total of 216,650,724 sequences reads for the csRNA library, 285,123,189 for the TEX library and 303,643,671 for the control library.

The reads were aligned to the reference genome of *E. histolytica*, using Bowtie version 0.12.7^63^ with the following parameters: maximum 2 mismatches were allowed (− n 2) and reads mapped to multiple locations (− m 50) were reported only once (− k 1). The produced alignments were sorted and indexed with SAMTools^59^. Coverage graphs representing the numbers of mapped reads per nucleotide were generated based on the sorted reads using BEDTools^64^, focusing on 5′ end position (− 5). On each coverage an upper quartile normalization^65^ was performed.

For each library, potential TSS were identified at the positions where all the following conditions were met (${e}_{L}i$ is the coverage at position i in the graph L):

- Minimum coverage: $$e_{TEX\left( + \right)|csRNA} i \ge 5$$
- Minimum ratio: $$\frac{{e_{TEX\left( + \right)|csRNA} i}}{{e_{Control} i}} \ge 1.5$$

TSS of both libraries were then merged, as the 2 methods were identified as consistent. TSS candidates within 10nts from each other were then clustered together in transcription initiation clusters and the position of the strongest coverage was defined as the peak.

Each TSS cluster was then classified as “*gene TSS”* (gTSS), an “*internal TSS”* (iTSS), an *“antisense TSS”* (asTSS), or an “*orphan TSS”* (oTSS) if it could not be assigned to any of the previous classes^33^. A TSS cluster was classified as gTSS if it was located ≤ 100 bp upstream of a gene and as asTSS if it was located within the 200 bp surrounding the stop codons on the antisense strand. The TSS cluster with the strongest expression values (maximum peak height) among gTSSs of a gene was classified as primary (pTSS). *“Internal TSS”* (iTSS) were located within an annotated gene on the sense strand. TSS belonging to none of these conditions (intergenic TSS) were annotated as *“orphan TSS”* (oTSS).

### PAS identification and annotation

First, reads with a stretch of five or more ‘A’ at their ends (or ‘T’ at their beginning) were selected for this analysis, as they potentially contain mRNA poly(A) tails. Redundant reads were removed and stretches of A at the ends were trimmed. Remaining reads with a minimum length of 18nt were then mapped on the reference genome using Bowtie^63^ with following parameters : − n 2 − k 1 − m 50 − l 30. To avoid false positives due to sequencing errors, reads with low quality (< 20) around PAS (5nt up and downstream) were removed from the set. To discriminate real poly(A) tracks of true polyadenylation from poly(A) tracks of internal homopolymeric stretches on the mRNAs, false positives were discarded with the following criteria: (1) reads with ≥ 8 nt within 10 nt immediate upstream of the PAS are A’s, (2) mapping with >  = 5 nt immediate downstream of the PAS are A’s.

PAS candidates within 12 nts from each other were then clustered together in PAS clusters and the position of the strongest coverage was defined as the peak. PAS with less than 2 reads of coverage at the peak were dismissed.

### Motif enrichment

The sequences immediate upstream and downstream of the gTSS, and asTSS (100nt on each side), as well as the PAS of mRNA and NAT (50nt on each side) were used to scan for conserved motifs using DREME^66^. The immediate upstream or downstream sequences were thus used as the positive sets, and the farther upstream (at position − 200) or downstream (at position + 150) sequences of the same length were used as the negative sets. To visually investigate the positional enrichment of these discovered motifs surrounding the polyadenylation sites, the total occurrence of these motifs was searched along the sequences surrounding (300 nt) the poly(A) sites.

### Differential expression analysis

Reads sequences of each replicate were mapped to the reference genome of *E. histolytica*, using STAR v2.5.0^58^) with a maximum intron length of 900 (–alignIntronMax), 2 mismatches maximum (–outFilterMismatchNmax), 10 locations maximum for a read mapping (–outFilterMultimapNmax), minimum overhang of 25 for spliced alignments.

Reads counting for each gene was produced by featureCounts^62^ separately on the direct (− s 1) and opposite strand (− s 2), allowing multi-mapping reads (− M). Normalization was first calculated on the sense counting, and size-factors were then applied to both sense and antisense counting. Transcript differential expressions were calculated on the merged normalized counting (sense and antisense) using DESeq2 v1.24.0^67^ within the SARTools pipeline v1.7.2^68^.

Analysis was conducted in R^69^ and figures were produced using the package ggplot2^70^.

The identification of protein classes corresponding to genes harboring an antisense transcript was performed with PANTHER tools (https://pantherdb.org).

## Supplementary information

Supplementary Information.

Supplementary Table 1.

Supplementary Table 2.

Supplementary Table 3.

Supplementary Table 4.

Supplementary Table 5.

Supplementary Table 6.

Supplementary Table 7.

Supplementary Table 8.

Supplementary Table 9.

Supplementary Table 10.

Supplementary Table 11.

Supplementary Table 12.

Supplementary Table 13.

## Data Availability

The data from experiments of TSS identification and heat-treated amoebae (dataset 2 and 3 in this paper) are available in the SRA database (https://www.ncbi.nlm.nih.gov/sra/) under the accession number PRJNA615171. The data concerning virulence conditions (dataset 1) were already in the ArrayExpress database (www.ebi.ac.uk/arrayexpress) under accession number E-MTAB-4882.
