# Supplementary material for: Genomic determinants for initiation and length of natural antisense transcripts in Entamoeba histolytica
Source: Sci Rep. 2020 Nov 19;10:20190. doi: 10.1038/s41598-020-77010-4 (PMC7677554; doi:10.1038/s41598-020-77010-4)
Supplement: Supplementary file 1 — Supplementary Information. [file 41598_2020_77010_MOESM1_ESM.pdf]

Supplementary information

Genomic determinants for initiation and length of natural antisense transcripts in *Entamoeba histolytica*

Damien Mornico[§4*], Chung-Chau Hon[§1,2#], Mikael Koutero[3], Christian Weber[1,2], Jean-Yves Coppee[3], Marie-Agnes Dillies[4] and Nancy Guillen[1,2,5*]

1 Institut Pasteur, Unité Biologie Cellulaire du Parasitisme, Paris, France;

2 Institut National de la Santé et de la Recherche Médicale, INSERM U786, Paris, France;

3 Institut Pasteur, Plate-forme Transcriptome et Epigénome, Paris, France;

4 Institut Pasteur, Hub de Bioinformatique et Biostatistique – Département Biologie Computationnelle, CNRS USR 3756, Paris, France;

5 Centre National de la Recherche Scientifique, CNRS ERL9195, Paris, France.

A

1,400 bp at contig DS571358. gene EHI_036570. CDS 1152 nt

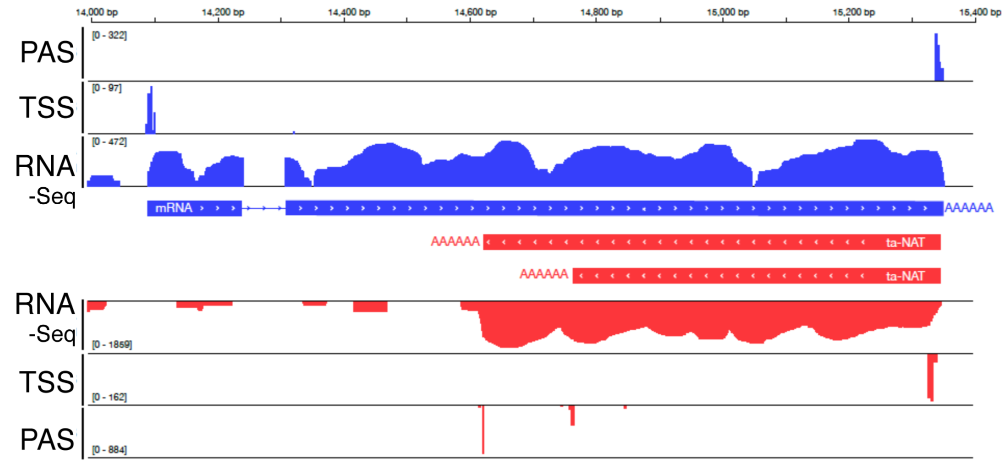

B

```
>EHI_036570A

ATGGATCAAATAGAACAAGCTATTAGTACTATCAAAAATGAGTATATCAATGCGAGTTCA
ATTGAAGATATTGTTGAGATATTGAAACGTACAATTATGATACTTGATGATATGAATAAA
AATAATAAAATAACAGAGAAAGAACAATTTGATATAATGATTCAAGATATTCTTAATTTG
ACTAGTAAAAAGAAAAGTACATTATATCTTGTTAAAAATGTTGTAACGGAAAAACTAAGC
AAAGAAATTAAAAAACGCTATGATATAACTGAAGAAGACATTAAAACTATGAAAGAACTA
TACAACCACAATGACTCAGACAATATATTATCTGTTGAAGAAATTGCTAAACGAATTAAT
GAAATGAGTCAAGAAGATTTTGAAACATTTATTGAATTAGAAAAAGGAAGAATAAATGAA
AAAACTATTTGTAAAGCTGTTCAAAATGAGTTAATTGAATCAAAAGAACGAGCGGTCTAC
ATTAGAATTGGGTTTCAACAAACATTTACTTCACTTGAAGTAAAGAAAGTTGAAGGGTTA
GTTTCAAAAATTAATAAAAAAGTATCAACACAAAATGTATTGAAAGGAACAGGAATTAAA
ATGAGACTATCAGCTGAGTTAAGAGACATTGGGATTTATAGTCATTTAGAACAATTAAAC
AAATGGACAAAAACAAAAGGAGCTGATTTAATATTTGATTCAACCTGTGATAAATATGAA
ACATTGATGAACACTGTCAGTGGAAAGAAACGATTAATGTATATTGTTGAAACAAATGAT
AATAATGTTTTTGGTACATTTAATGGAATGAGTCTGAATAATTATGGAGACAAAATTACA
AATGACACAAAACATTTCATGTTCTCTATTATTAATCCAAGTAAAACAGAACCATTAATG
TTTTCTATGAAACTAAAAGTAATAGACAGTTTATGTATTGATTCCAAAGATGGAAAAATA
ATTACATCATTAGGATGTTGCACTCTTGTTGCAGATACAGATGATGATAATCCTAATGTA
GCAGGAACAATTGATGGACATTTTGGAGAATACTATAAAGATAAAAAAGGGTATGGAGCA
AAAATATTTGATGGGAAAGTAAATCCACAAGTATTTAATGTGAAAAAATTGTTGGTGCTT
GAAATGAAATGA

PCR1: DNA fragment of 194bp for antisense detection (probe A)

EHI_36570 Forward: T7 primer + 5'CTGTTCAAAATGAGTTAATTGAATCA
EHI_36570 Reverse: ------------5'CCTGTTCCTTTCAATACATTTTG
reversed and complemented from 5'CAAAATGTATTGAAAGGAACA


PCR2: DNA fragment of 565bp for antisense detection(probe B)

EHI_36570 Forward: T7 primer + 5'TGAAAGGAACAGGAATTAAAATGAGAC
EHI_36570 Reverse: ------------5'TTTGCTCCATACCCTTTTTTATC
reversed and complemented from 5'GATAAAAAAGGGTATGGAGCAAA

T7 primer: 5'TAATACGACTCACTATAGGGAGA
```

C

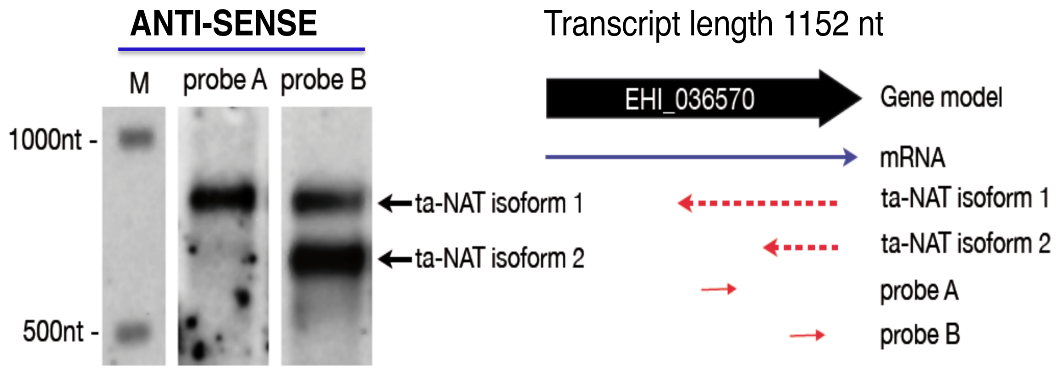

D

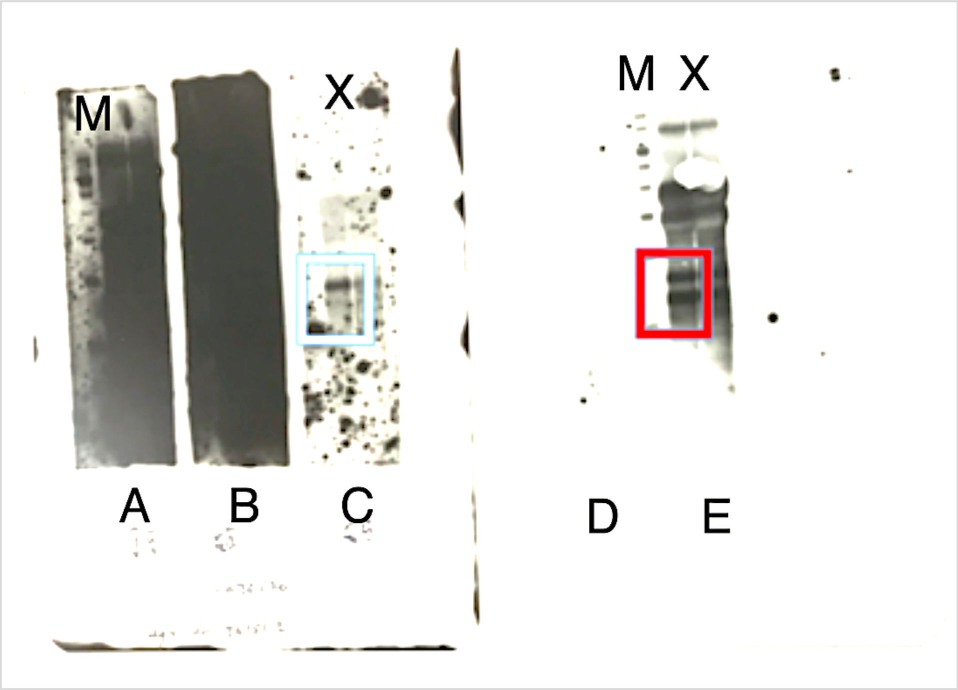

Supplemental Figure 1. Northern Blot to detect NATs.

A. The gene EHI_036570 (CDS of 1152 nt) was chosen for NATs detection. According to the bioinformatic analysis this gene present two NATs.

B. Two biotinylated probes were constructed according to the sequences underlined for PCR1 and PCR2 and submitted to in vitro transcription under the control of T7 polymerase.

C. In the blot, probe A recognizes the NAT of higher size (roughly of 750nt plus the poly(A) tail of unknown length) and probe B recognizes the two NATs (same that these of probe A and another of 600nt with the poly(A tail of unknown length). The white delineation between the tracks was used to separate the data obtained with the same blot hybridized with different probes. Radiolabeled size standards are in the left side (M).

D. Original autoradiogram corresponding to the Northen blot : the lanes with the lower RNA concentration are indicated with X and the area within the squares were taken to prepare the panel C in this figure. Different quantities of RNA were loaded and hybridized with probe A (A,B,C) or with probe B (D, E). Lines M correspond to the marker of RNA length at 500nt and 1000 nt

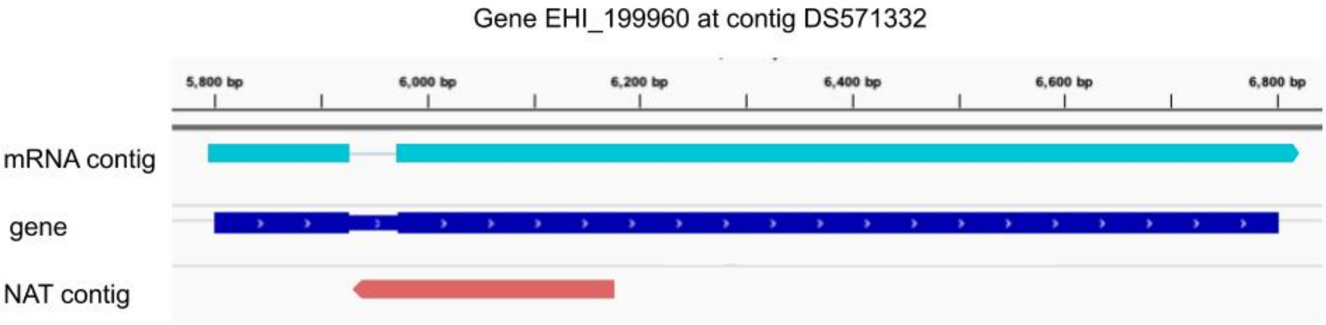

Supplemental Figure 2. NAT contig overlapping gene intron. Integrative Genome Viewer (IGV) screenshot of gene EHI_199960 presenting mRNA and NAT contigs with an overlapping between intron and NAT.

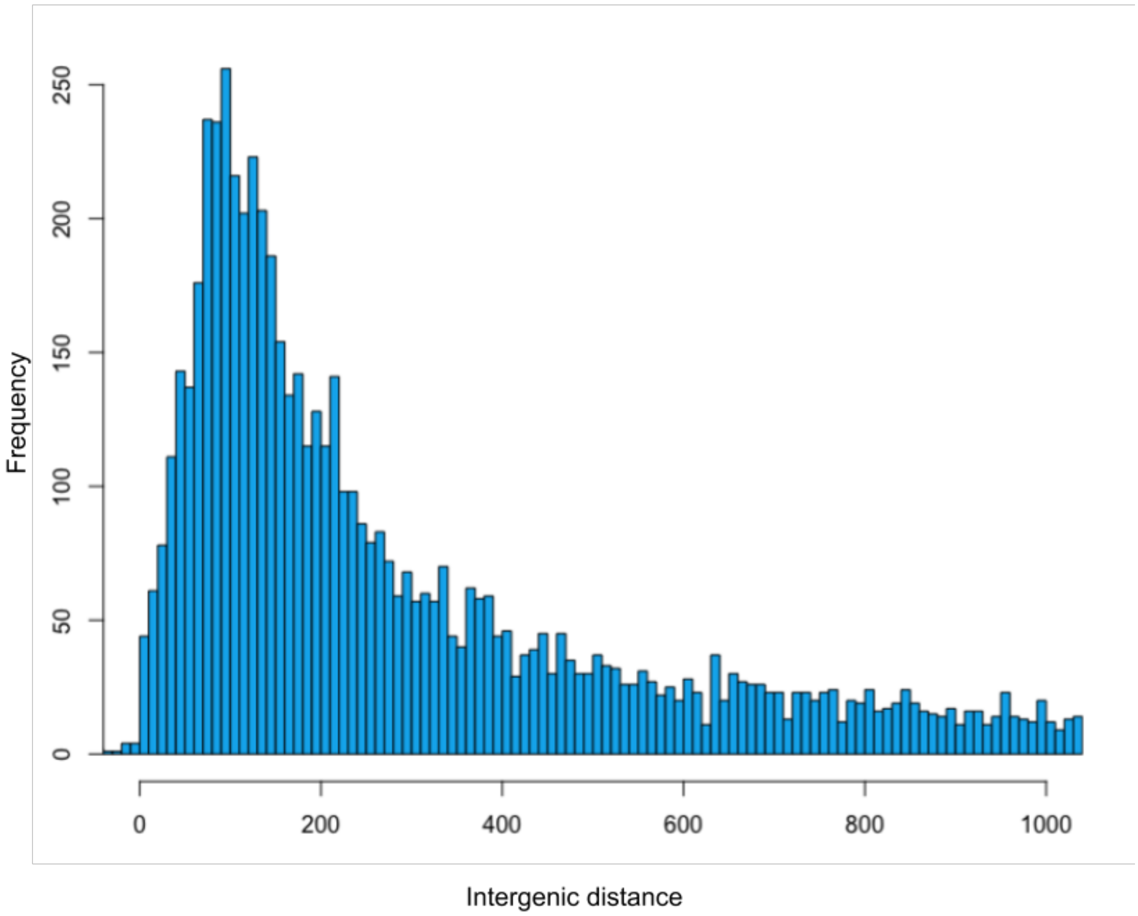

Supplemental Figure 3. Intergenic distance distribution. Showing a peak at 88nt and a median at 250nt.

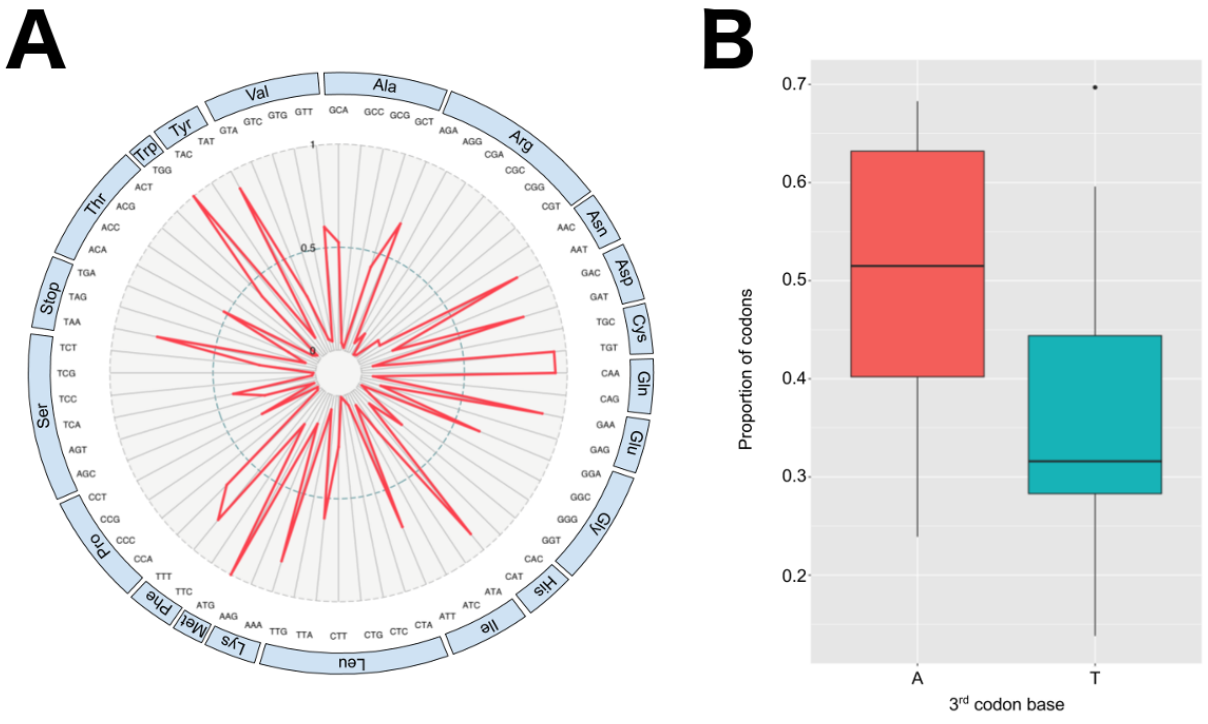

Supplemental Figure 4. Proportion of synonymous codons with a T or a A at the 3rd position.

(A) For each amino-acids, proportion of the given codons have been measured in genes with NAT PAS

(B) For codons leading to the same amino-acid, proportion of A as third base is higher than T, allowing greater frequencies of U-rich motifs on the opposite strand.
